# Melatonin Alleviates Drought Stress by a Non-Enzymatic and Enzymatic Antioxidative System in Kiwifruit Seedlings

**DOI:** 10.3390/ijms21030852

**Published:** 2020-01-28

**Authors:** Hui Xia, Zhiyou Ni, Rongping Hu, Lijin Lin, Honghong Deng, Jin Wang, Yi Tang, Guochao Sun, Xun Wang, Huanxiu Li, Mingan Liao, Xiulan Lv, Dong Liang

**Affiliations:** 1 College of Horticulture, Sichuan Agricultural University, Chengdu 611130, China; 2 Institute of Plant Protection, Sichuan Academy of Agricultural Sciences, Chengdu 610066, China

**Keywords:** melatonin, drought stress, carotenoid, ascorbic acid, kiwifruit

## Abstract

Although melatonin was affirmed to alleviate drought stress in various plant species, the mechanism in kiwifruit remains to be elucidated. In this study, the transcriptomes of kiwifruit leaves under control (CK), DR (drought stress), and MTDR (drought plus melatonin) treatments were evaluated. After comparisons of the gene expression between DR and MTDR, the differentially expressed genes (DEGs) were screened. Kyoto Encyclopedia of Genes and Genomes (KEGG) enrichment analyses indicated three significant pathways, which were mainly involved in the glutathione metabolism, ascorbate and aldarate metabolism, and carotenoid metabolism. Therefore, the content and metabolic gene expression level of ascorbic acid (AsA), glutathione, and carotenoid were higher in the MTDR treatment than that in others. Furthermore, the activity and mRNA expression level of superoxide dismutase (SOD), catalase (CAT), and peroxidase (POD) were also promoted in the MTDR group. Combined with these results of important secondary metabolites and protective enzymes measured in the seedlings in different treatments, it could be concluded that exogenous melatonin induced the ascorbic acid-glutathione (AsA-GSH) cycle, carotenoid biosynthesis, and protective enzyme system to improve seedling growth. Our results contribute to the development of a practical method for kiwifruit against drought stress.

## 1. Introduction

Drought is becoming a fateful environmental challenge and occurs in many regions every year in the world [1,2]. As water is indispensable for plants, water scarcity has negative impacts on their growth and agricultural productivity [1,2]. Drought stress causes a reduction in the hydration of the membranes and proteins, thus leading to the damage of these components and accompanied by the excessive accumulation of reactive oxygen species (ROS), such as O_2_^−^, H_2_O_2_, ^1^O_2_, HO_2_^−^, OH·, ROOH, and ROO [3]. These ROS cause damage to chloroplast and mitochondria and degenerate the cellular structure due to their high reactivity and toxicity [4,5].

Kiwifruit is one of the most popular fruits worldwide, and is impressively rich in vitamin C and mineral element contents, as well as an increasing world market [6]. Kiwifruit has generally evolved in high-humidity areas and is naturally intolerant to drought stress. Under drought stress, levels of malondialdehyde (MDA) and H_2_O_2_ increase continuously, and lowers leaf water potential, photosynthetic rate, and stomatal conductance in kiwifruit leaves [7,8]. Therefore, water deficits in the growing season can negatively affect the growth and productivity of kiwifruit, which becomes a key bottleneck in the development of the kiwifruit industry in the world [9].

As a highly conserved molecule in evolution, melatonin (N-acetyl-5-methoxytryptamine) has widely existed in species from bacteria to mammals [10]. In plants, melatonin plays critical functions in regulating plant growth and development, including vegetative growth promotion, seed germination, rooting, flowering, and senescence [11]. Meanwhile, research has found that melatonin acts as a potent free radical scavenger, thus reducing the toxicity of a wide variety of environmental stresses, including drought, heavy metals, salinity, UV radiation, extreme temperatures, and pathogens, in various plants [12]. Lots of studies have investigated melatonin-mediated regulation of plant biology in response to drought stress, such as in oat (*Avena nuda*) [13], apple (*Malus domestica*) [14,15], cucumber (*Cucumis sativus*) [16], grape (*Vitis vinifera*) [17], *Malus prunifolia* [18], tomato (*Solanum Lycopersicum*) [19,20], maize (*Zea mays*) [21,22], wheat (*Triticum aestivum*) [23], creeping bentgrass (*Agrostis stolonifera*) [24], kiwifruit (*Actinidia chinesis)* [25], and coffee (*Coffea arabica)* [26]. The main conclusions of these studies include the following four aspects: (1) Melatonin can protect the photosynthetic apparatus and improve the photochemical efficiency of photosystem II, which promotes the photosynthetic capacity response to the deleterious effects of drought [14,15,16,17,19,20,22,23,25,26]; (2) melatonin also protects plants against drought-induced oxidative stress by enhancing the ROS scavenging efficiency. The intensive ROS scavenging capacity stems from the melatonin-stimulated enzymatic and non-enzymatic antioxidative defense system under drought conditions, for example, superoxide dismutase (SOD), catalase (CAT), peroxidase (POD), ascorbic acid-glutathione (AsA-GSH) cycle, and osmolytes [14,15,16,17,19,20,22,23,25,26]; (3) melatonin induces the transcriptional level of the mitogen-activated protein kinase (MAPK) signaling pathway and its main components (transcription factors, including WRKY, DREB, MYB, and NAC) upregulation, which are responsible for drought stress tolerance [13,24,27]; and (4) melatonin provides drought tolerance by regulating phytohormone metabolism pathways. Melatonin promotes drought stress tolerance by suppressing indoleacetic acid (IAA) biosynthesis via GA signaling, stimulating cytokinin (CK) biosynthesis, and reducing abscisic acid (ABA) accumulation [18,21,24,27,28]. The results of our previous study indicated that exogenous melatonin can effectively improve the repression of biomass accumulation and photosynthesis caused by drought stress [25]. Despite these results, whether melatonin possesses the same functional mechanism or other discovered roles for kiwifruit against drought stress remains unknown.

To survey the molecular mechanism of how melatonin modulates the kiwifruit response to drought stress, here, we characterized the global gene expression patterns for kiwifruit seedlings from control (CK), drought (DR), and melatonin plus drought (MTDR) treatments via RNA-sequencing (RNA-Seq). Large sets of differentially expressed genes and enriched Kyoto Encyclopedia of Genes and Genomes (KEGG) pathways were identified between comparisons. Furthermore, the expression patterns of key genes and production concentrations involved in the objective KEGG pathways were characterized. The present work provides valuable insights into the mediation of melatonin in mechanisms underlying the drought responses of kiwifruit plants.

## 2. Results

### 2.1. Morphologic and Physiologic Responses to Drought Treated by Melatonin

**Figure 1:**
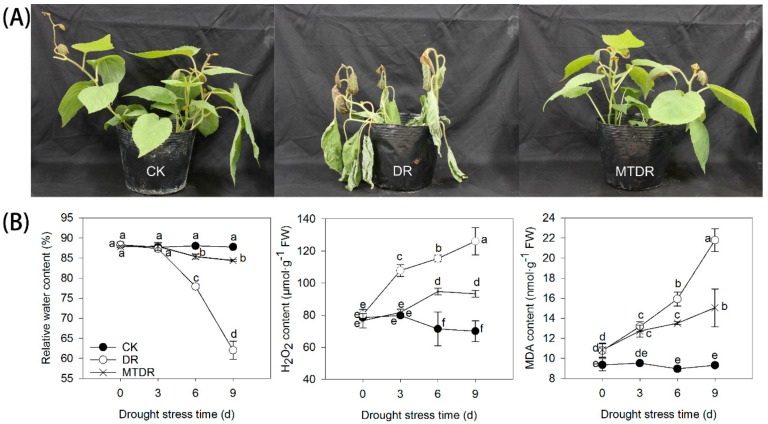
Effects of melatonin pretreatment on the plant phenotype (**A**), leaf relative water content, H_2_O_2_ content, and MDA content (**B**) of kiwifruit seedlings exposed to drought. CK, samples well-watered; DR, samples pretreated with water, subsequently stressed by drought for 9 days; MTDR, samples pretreated with 100 µM melatonin solution, subsequently stressed by drought for 9 days. Different letters indicate significant differences according to LSD tests (*p* < 0.05).

With irrigation continually withheld for 9 days, the leaves of kiwifruit seedlings (DR) were gradually dehydrated and ultimately wilting (Figure 1A), and the relative water content in leaves decreased from 80.23% on 0d to 56.40% after 9 days (Figure 1B). Meanwhile, the content of H_2_O_2_ in leaves increased rapidly from 80.30 µmol/g FW on 0 days up to 126.05 µmol/g FW), and the MDA content increased from 10.77 to 21.78 nmol/g FW on 9 days (Figure 1B). Apparently, pretreatment with melatonin immensely alleviated these symptoms, as plants pretreated with melatonin (MTDR) exhibited a similar growth status with seedlings under optimum conditions (CK), with a 76.71% relative water content, 93.37 µmol/g FW H_2_O_2_ content, and 15.05 nmol/g FW MDA in leaves on 9 days, respectively (Figure 1B).

### 2.2. Transcriptome Profiling and Quality

To develop a comprehensive overview of the effect of melatonin on kiwifruit seedlings under drought stress, expression profiles of three different treatments (CK, DR, and MTDR) on 9 days were assayed using RNA sequencing. After removing low-quality reads, a total of 45,707,847 (CK), 46,895,636 (DR), and 51,348,163 (MTDR) clean reads were acquired from the kiwifruit leaves, respectively, corresponding to 6.86, 7.04, and 7.71 G. Additionally, more than 97.12% of the reads had a quality score of Q20 (sequencing error rate, 0.01%). Over 67.71% of the total reads were mapped to the kiwifruit reference genome, and uniquely mapped reads ranged above 65.18% (Table 1).

### 2.3. Gene Expression Analysis

**Figure 2:**
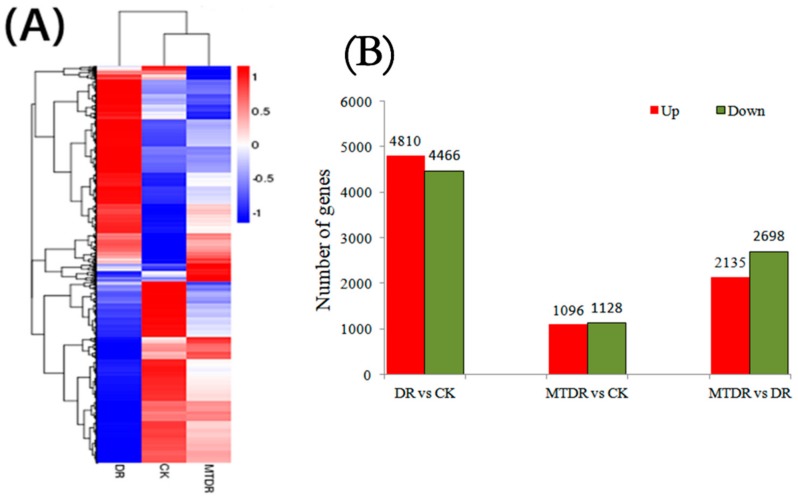
The differentially expressed genes (DEGs) analysis by the hierarchical clustering heat map (**A**) and number of regulated genes in the different comparisons (**B**). (**A**): the color scale in the heat map ranged from blue to red, representing log_10_ (FPKM+1) of −1 to 1. (**B**): red and green columns represent genes with significantly different expression.

To observe the gene expression pattern as a whole, we made a hierarchical clustering of the differentially expressed genes based on the three samples’ log_10_ (FPKM+1), with an adjusted *p*-value < 0.05 (Figure 2A). The results showed a starkly different gene expression pattern compared to CK with DR, while MTDR presented a similar expression pattern with CK, indicating that the application of melatonin before a water deficit had a significant influence on the global gene expression profile of kiwifruit leaves under drought stress.

Furthermore, differentially expressed genes (DEGs) specific to the samples were performed at a statistically significant value (corrected *p*-value < 0.05). A total of 9276 DEGs were identified between CK and DR, with 4810 upregulated and 4466 downregulated genes. There were a total of 2224 DEGs identified from MTDR versus CK, including 1096 upregulated genes and 1128 downregulated genes. In total, 2135 upregulated and 2698 downregulated genes were detected when compared to MTDR with DR (Figure 2B). Notably, more genes were differentially expressed in DR vs. CK than those in MTDR vs. CK, suggesting that non-melatonin treatment could affect drought stress-related genes more than melatonin pretreatment.

### 2.4. Verification of RNA-Seq Data by qRT-PCR

**Figure 3:**
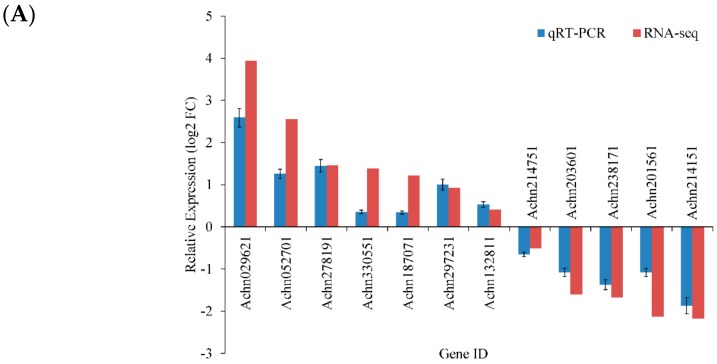

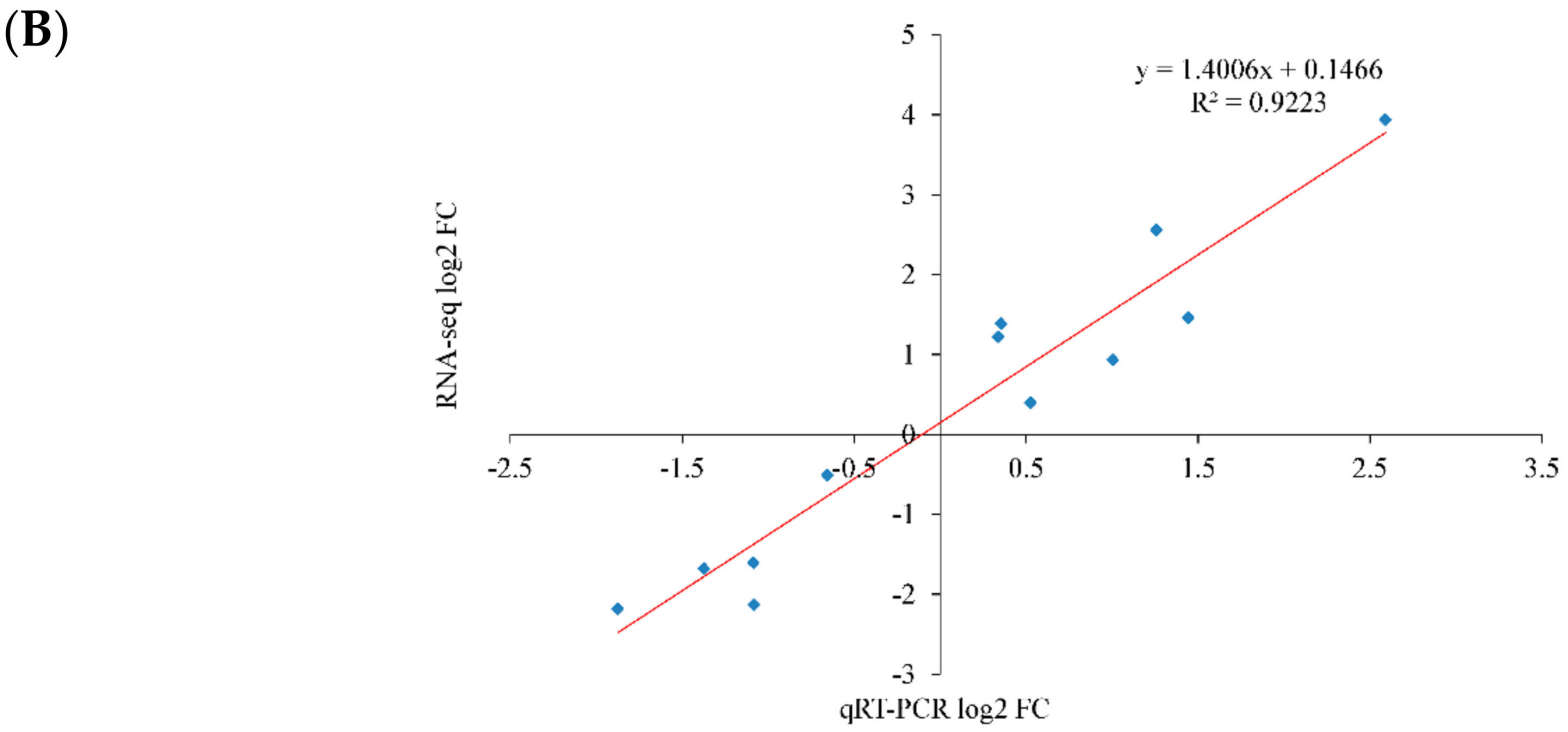
Expression pattern of 12 selected DEGs from MTDR vs. DR as obtained by RNA-seq and qRT-PCR. (**A**) Histogram of 12 selected genes based on the fold change measured by RNA-seq and by qRT-PCR analysis of MTDR and DR, respectively. (**B**) A linear trend line is shown with Pearson’s correlation to determine the relationship between the qRT-PCR and RNA-seq results for DEGs expression levels. FC is the ratio of FPKM between MTDR and DR.

To confirm the accuracy and reproducibility of the Illumina RNA-Seq results, 12 genes were chosen from DEGs in the DR and MTDR libraries for quantitative real-time PCR detection. Nearly all of these genes presented a similar expression trend in both techniques (Figure 3A). Additionally, the correlation between qRT-PCR and RNA-Seq was measured by a scatter plot of log_2_ (MTDR/DR) and log_2_ (FC) (Figure 3B), which showed a positive correlation coefficient (Pearson’s correlation coefficient R^2^ = 0.92). As a result, the qRT-PCR results for the selected genes displayed great agreement with their transcript abundance changes as determined by RNA-Seq, although different algorithms were determined by the two techniques, suggesting that the data obtained from RNA-Seq analysis were credible.

### 2.5. Function Classification of DEGs

To identify whether the drought stress-responsive genes engaged in specific pathways, the DEGs were assigned to the reference canonical pathways in KEGG. In our analysis, glutathione metabolism, ascorbate and aldarate metabolism, and carotenoid metabolism were significantly enriched, to which some of the upregulated DEGs from MTDR vs. DR were mapped (Table 2). These pathways provide a valuable resource for investigating specific processes, functions, and pathways in the role of melatonin played in kiwifruit seedlings’ drought stress research.

### 2.6. Effects of Melatonin Pretreatment on AsA Metabolism

**Figure 4:**
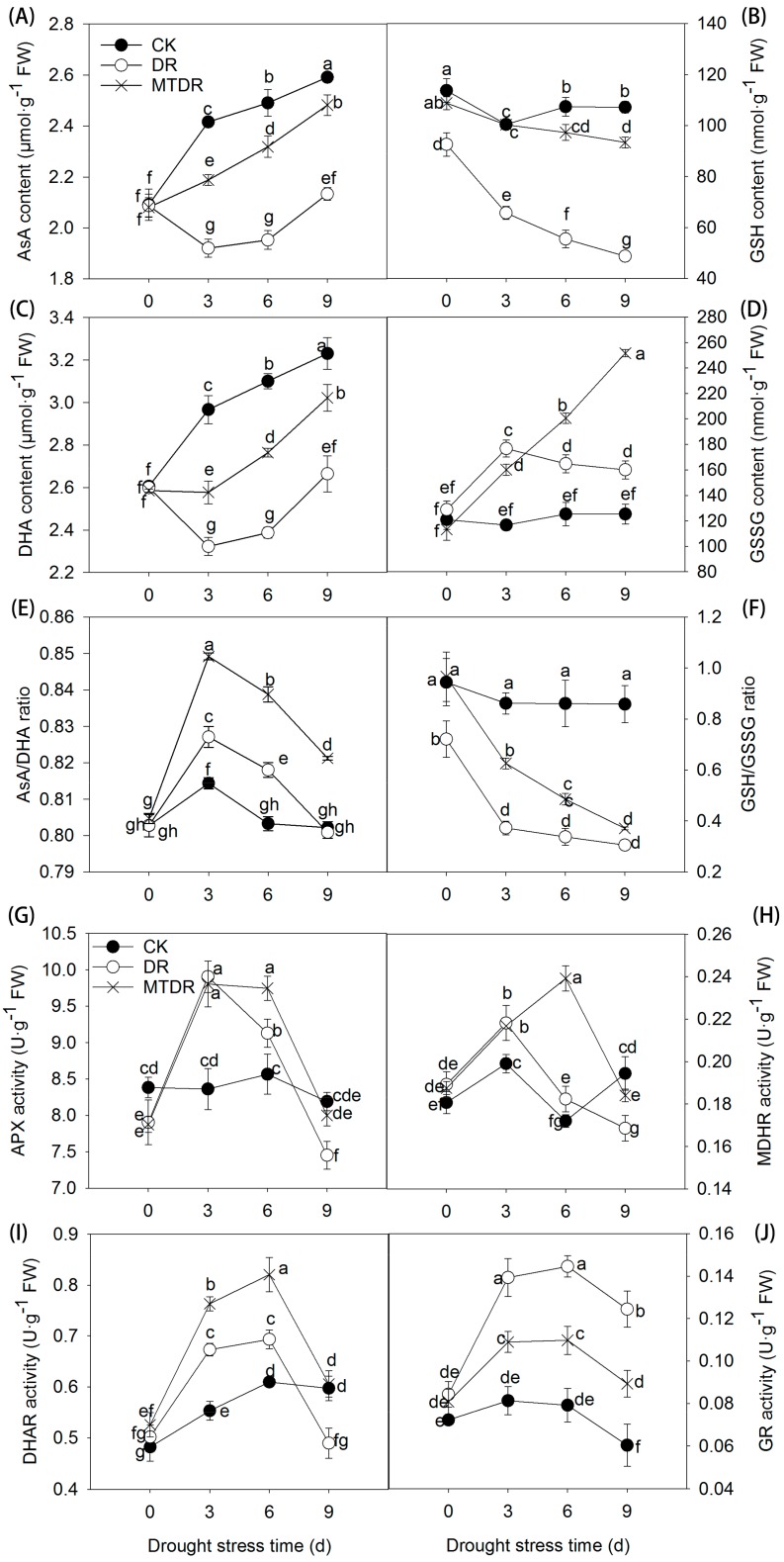
Effects of melatonin pretreatment on the AsA-GSH cycle of kiwifruit seedlings exposed to drought. (**A**) AsA content, (**B**) GSH content, (**C**) DHA content, (**D**) GSSG content, (**E**) AsA/DHA ratio, (**F**) GSH/GSSG ratio, (**G**)APX activity, (**H**) MDAR activity, (**I**) DHAR activity, and (**J**) GR activity. Data represent means ± S.D. of at least three replicate samples. Different letters indicate significant differences according to LSD tests (*p* < 0.05).

AsA and GSH are of great importance in maintaining the cellular redox balance. In our study, reduced AsA and DHA contents performed the same trend under drought stress, which slightly decreased on 3 days and then gradually increased (Figure 4A,C). On the contrary, the oxidized glutathione (GSSG) content showed a peak on 3 days, while GSH decreased in the whole drought stress process (Figure 4B,D). Obviously, the application of melatonin improved the concentrations of reduced AsA, and also mitigated the decline of reduced GSH, which increased by 16.35% and 91.49% compared with untreated seedlings on 9 days, respectively. Besides, DHA and GSSG were correspondingly improved by melatonin. The ratios of AsA/DHA and GSH/GSSG function as signals for the regulation of antioxidant mechanisms. Here, a significant AsA/DHA ratio burst occurred on 3 days (Figure 4E), whereas the GSH/GSSG ratio tended to vary differently, which decreased all the time (Figure 4F). Similarly, melatonin application maintained the two ratios at a higher level compared with untreated plants under drought stress.

The four enzymes, ascorbate peroxidase (APX), monodehydroascorbic acid reductase (MDAR), dehydroascorbic acid reductase (DHAR), and glutathione reductase (GR), involved in the AsA-GSH cycle presented similar trends, with activity increasing initially and afterwards decreasing. The APX and MDAR activities both showed a peak on 3 days under drought stress, then falling, whereas, melatonin application caused a summit of APX and MDAR on 3 and 6 days, respectively. APX activity was higher for the next 3 days (Figure 4G,H). The highest value of DHAR and GR activities appeared on 6 days in both the drought and melatonin treatment (Figure 4I,J). Moreover, the application of melatonin accelerated the DHAR activity and showed a greater level than that of the drought treatment. In contrast, the GR activity of melatonin-pretreated seedlings was lower than that of untreated ones.

**Figure 5:**
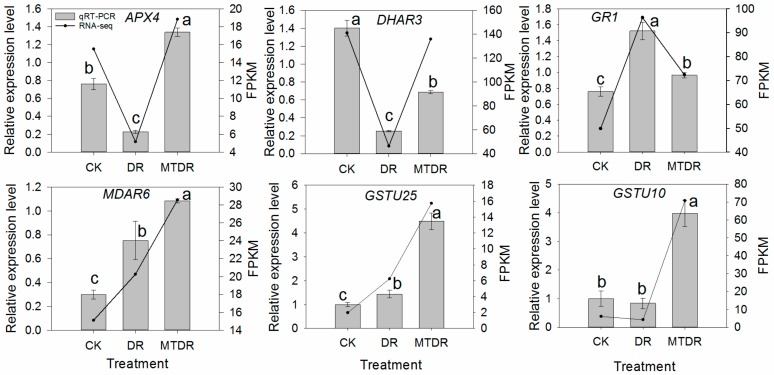
Expression pattern of genes involved in AsA metabolism by RNA-Seq and qRT-PCR exposed to drought at 9 days. Different treatments (x-axis) and gene expression (y-axis) by FPKM (right) and relative expression levels (left). Gray columns in all plots indicate the relative expression level obtained by qRT-PCR; the black lines indicate the FPKM value obtained by RNA-seq. Bars represent the standard error (*n* = 3). Different letters indicate significant differences according to LSD tests (*p* < 0.05) for qRT-PCR.

The transcription of 16 DEGs on 9 days involved in AsA metabolism was investigated (Figure 5 and Supplementary Material 1). For the AsA-GSH cycle, *APX4*, *APX6*, and *AO* (ascorbate oxidase), all presented higher expression levels in MTDR than those in DR. Similarly, *DHAR* was expressed more in MTDR than that in DR. Although *MDAR4* and *MDAR6* displayed different expression patterns, they were the highest in MTDR. In contrast, the transcription of *GR1* in MTDR was lower than that in DR (Figure 5 and Supplementary Material 1). Regarding AsA biosynthesis genes, including gene *GME* (GDP-mannose 3,5-epimerase), *VTC2* (GDP-L-galactose phosphorylase), *MIOX4*, and *MIOX5* (myo-inositol oxygenase), and genes *GSTL2*, *GSTU10*, *GSTU18*, *GSTU19*, and *GSTU25* (glutathione-S-transferase), all of them were upregulated when comparing MTDR and DR (Figure 5 and Supplementary Material 1).

### 2.7. Effects of Melatonin Pretreatment on Carotenoid Metabolism

**Figure 6:**
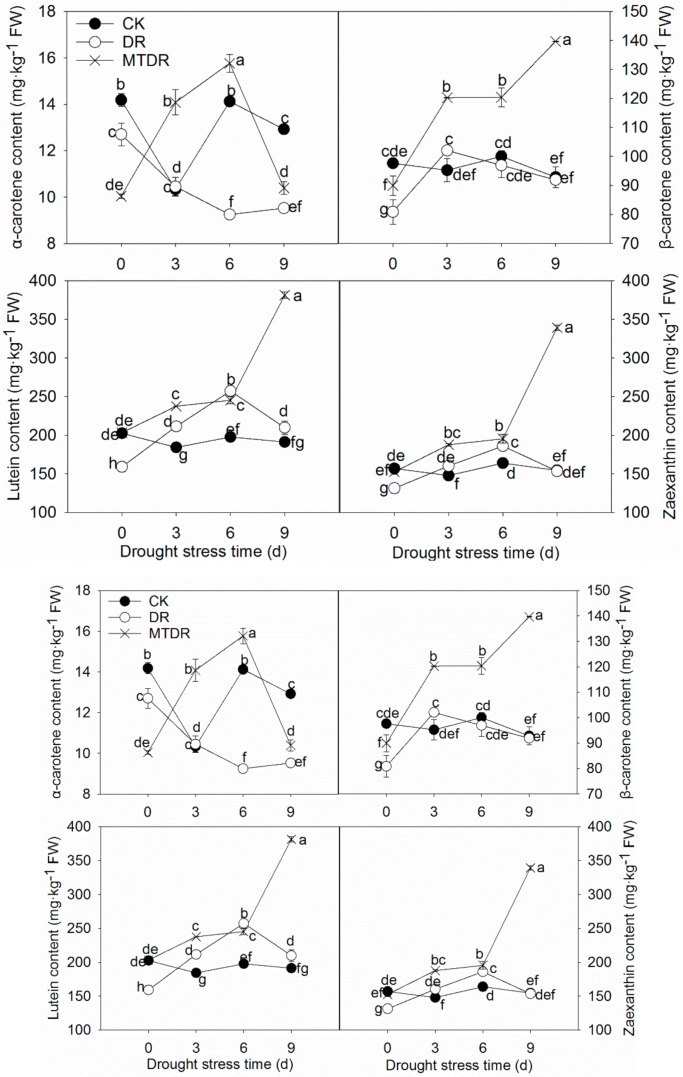
Carotenoid content profiles of kiwifruit seedlings exposed to drought. Data represent means ± S.D. of at least three replicate samples. Different letters indicate significant differences according to LSD tests (*p* < 0.05).

The contents of four carotenoid components, α-carotene, β-carotene, lutein, and zeaxanthin, were determined by HPLC (Figure 6). Compared with the α-carotene content of DR, which was in a state of decline, the α-carotene content of MTDR was significantly higher during drought stress, although it dropped on 9 days. Similar β-carotene, lutein, and zeaxanthin content trends were observed (Figure 6). All of them increased firstly and subsequently reduced under drought stress, while priming with melatonin promoted their accumulation. After the 9-day water withholding, the α-carotene, β-carotene, lutein, and zeaxanthin contents of MTDR were 9.14%, 51.94%, 81.64%, and 120.96% higher than those of DR, respectively.

**Figure 7:**
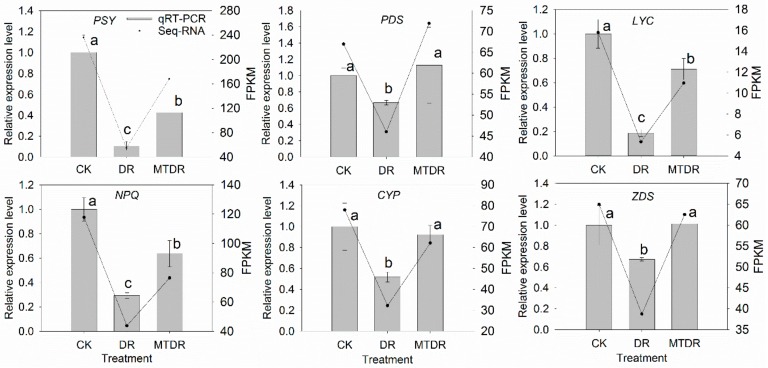
Expression profiles of genes involved in carotenoid metabolism in kiwifruit seedlings exposed to drought at 9 days. Expression pattern obtained by RNA-seq and qRT-PCR. Different treatments (x-axis) and gene expression (y-axis) by FPKM (right) and relative expression level (left). Gray columns in all plots indicate the relative expression level obtained by qRT-PCR in 9 days; the black lines indicate the FPKM value obtained by RNA-seq. Bars represent the standard error (*n* = 3). Different letters indicate significant differences according to LSD tests (*p* < 0.05) for qRT-PCR.

Moreover, six DEGs were annotated as key genes encoding enzymes related to carotenoid biosynthesis, including phytoene synthase (*PSY*), phytoene desaturase (*PDS*), lycopene beta cyclase (*LYC*), violaxanthin de-epoxidase (*NPQ1*), cytochrome P450 (*CYP*), and zeta-carotene desaturase (*ZDS*). All of them performed analogous expression patterns, and their transcription in MTDR was higher than that in DR (Figure 7).

### 2.8. Effects of Melatonin Pretreatment on Antioxidant Enzymes

**Figure 8:**
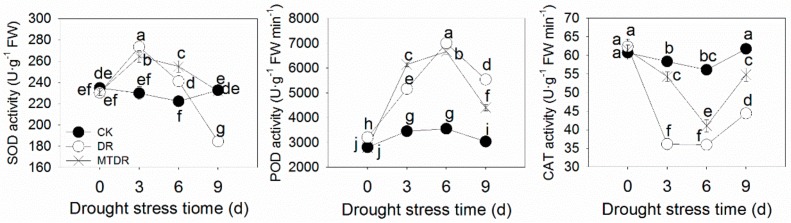
Antioxidant enzymes activities in kiwifruit seedlings exposed to drought. Data represent means ± S.D. of at least three replicate samples. Different letters indicate significant differences according to LSD tests (*p* < 0.05).

**Figure 9:**
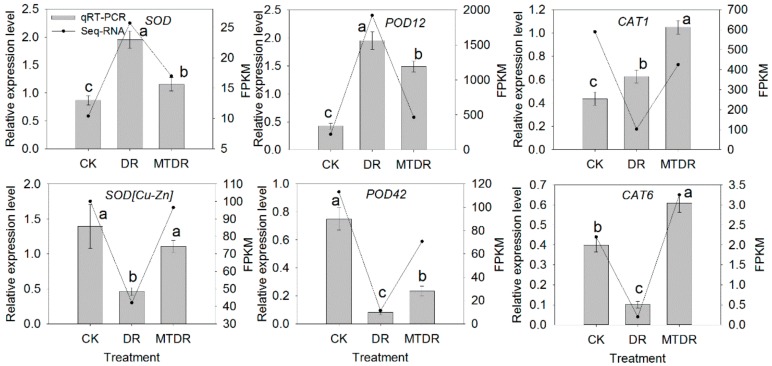
Antioxidant enzyme-related gene expressions in kiwifruit seedlings exposed to drought at 9 days. Expression pattern obtained by RNA-seq and qRT-PCR. Different treatments (x-axis) and gene expression (y-axis) by FPKM (right) and relative expression level (left). Gray columns in all plots indicate the relative expression level obtained by qRT-PCR in 9 days; the black lines indicate the FPKM value obtained by RNA-seq. Bars represent the standard error (*n* = 3). Different letters indicate significant differences according to LSD tests (*p* < 0.05) for qRT-PCR. Expression profiles of genes involved in carotenoid metabolism in kiwifruit seedlings exposed to drought for 9 days.

We assayed the activities of three antioxidant enzymes, SOD, POD, and CAT, which are involved in the process of removing ROS under drought stress. With the stress time prolonged, SOD activity increased on 3 days and afterwards decreased gradually, and POD activity reached the summit on 6 days (Figure 8). However, the activity of CAT presented the opposite trend, with a decreasing value until 6 days and increasing later (Figure 8). With melatonin pretreatment, SOD activity in the later stage and POD activity in the early stage was apparently higher than that of untreated plants under drought stress. Additionally, CAT activity was significantly improved by melatonin application, which increased by 23.42% on 9 days compared with that of untreated seedlings. Overall, the decrease of the enzyme activities of SOD and CAT was effectively alleviated by melatonin during the drought stress process. In addition, the expression levels of the three antioxidant enzymes’ corresponding DEGs were determined (Figure 9) in 9 days. Apart from *SOD* and *POD12*, whose transcriptions in DR were the highest, *SOD [Cu-Zn]*, *POD42*, *CAT1*, and *CAT6* were expressed more in MTDR than those in DR (Figure 9).

## 3. Discussion

Currently, the changing climate scenario of global warming indicates an increase in drought episodes and severity in regions prone to drought. Thus, developing adaptive agricultural strategies is urgently needed [29]. In this study, 100 µM exogenous melatonin was used to irrigate kiwifruit seedlings under drought conditions, which were analyzed on the transcriptomic and physiological levels. The results showed that the kiwifruit seedlings’ drought resistance was significantly improved by melatonin due to an enhancement of the antioxidant ability, which has been considered the primary function of melatonin in plant stress tolerance.

Continuous drought stress induces a water deficit inside plant tissues, and damage to plants is always first reflected in the leaves which curl and fold gradually; further affects are shown on the physiological growth [30]. Since seedlings lost water gradually, the leaves exposed to drought began to turn yellow and roll up (Figure 1A). The seedlings pretreated with melatonin were less affected, and were closer to the normal growth state from the appearance (Figure 1A). The relative water content of leaves in the MTDR group decreased to a lesser extent than that of the CK group as the time of water deficit was prolonged but was higher than that of the DR group (Figure 1B). This meant that melatonin played a role in preventing moisture loss in kiwifruit plant leaves. Other studies on soybean [31] and wheat [23] have also shown that exogenous melatonin enhances plant maintenance of high leaf water retention capacity under dehydration conditions. In *Arabidopsis thaliana*, the results suggest that melatonin can regulate the stomatal closure by its receptor PMTR1 [32]. Melatonin-treated *Malus* species seedlings had significantly longer and wider stomata under drought stress when compared with those of the controls; however, stomatal density was less than that in the control [18]. Also, melatonin regulated the water balance in mesophyll cells along with their turgor pressure in wheat against drought stress [23]. Hence, the main reasons behind the improved cell water holding capacity induced by melatonin in drought stress is that melatonin regulates the opening/closure of stomata and turgor pressure, while the specific mechanisms are different in diverse species.

Drought stress triggers ROS accumulation and breaks down the balance between ROS generation and detoxification [17,31]. H_2_O_2_, one of the ROS, was accumulated a lot in the late stage of drought, indicating the presence of oxidative damage in the kiwifruit seedlings, which could lead to membrane lipid peroxidation, thus the MDA content synchronously increased. Appreciably, melatonin application significantly slowed the rising trend of the H_2_O_2_ and MDA concentrations, as shown in this work (Figure 1B), grape [17], and coffee [26], suggesting that the oxidative damage caused by drought were alleviated by melatonin as a result of the relatively lower ROS levels. This triggered ROS scavenging may be due the induced superoxide anions being controlled by melatonin [13,19]. Moreover, the scavenging efficiency is also enhanced by melatonin when plants grow under drought stress [16,18]. These results indicated direct ROS scavenging by melatonin.

Non-enzymatic antioxidants, such as carotenoids, polyphenols, AsA, and GSH, donate electrons to neutralize free radicals to non-reactive species. The high reactivity of free radicals results in the extraction of an electron from almost any available molecule, therefore the role of antioxidants is to protect all biomolecules from the oxidative damage caused by free radicals [5]. Carotenoids, including xanthophyll, α-carotene, β-carotene, lutein, and zeaxanthin, have a variety of crucial roles in photosynthetic organisms, such as photosystem assembly, enhancing light harvesting by absorbing a broader range of wavelengths than chlorophyll, providing protection from excess light via energy dissipation, and free radical detoxification [5]. Membrane-bound antioxidants, carotenoids can quench ^3^Chl and ^1^O_2_, inhibit lipid peroxidation, and stabilize membranes [33]. Thus, carotenoid accumulation in the leaves of kiwifruit in this study helps seedlings against drought stress (Figure 6), as found in eggplant [34] and pepper [35] in drought stress resistance. In addition, it is well known that abscisic acid (ABA) plays important roles in helping plants against different abiotic stresses. Carotenoids are not only the plant pigment but also a precursor for ABA biosynthesis [33], which may be another key reason why carotenoids accumulated in the DR and MTDR groups.

Another important antioxidant is AsA, which has a central role in preventing oxidative damage through direct quenching of _1_O^2^, O^2∙-^, and OH·, and is a substrate in APX reactions [5]. Moreover, GSH protects thiol groups in stromal enzymes, and is capable of detoxifying _1_O^2^ and OH· [5]. Both oxidized forms of AsA and GSH are relatively unstable in aqueous environments, while DHA can be chemically reduced by GSH to AsA, leading to the process of the AsA-GSH cycle. Melatonin mediated ROS scavenging induced by drought stress in plants via regulation of the key enzymes involved in AsA-GSH, as the activity of APX, DHAR, MDHAR, and GR increased in apple [14] and tomato [20] applied with exogenous melatonin under drought treatment. In the present study, the activity of APX, MDHAR, and DHAR in MTDR was higher than that in other treatments, which results in higher AsA/DHA and GSH/GSSG ratios (Figure 4). The enhanced ratios are considered as a vital mechanism for melatonin to alleviate drought stress [14,20].

One important function of melatonin may be to promote the activity of some antioxidant enzymes, such as SOD, POD, and CAT. Then, the enhanced activity of the above-mentioned enzymes results in scavenging of ROS, indicating an indirect mechanism [36]. Here, the activities of SOD, POD, and CAT in the MTDR groups exhibited higher values in different treated stages compared with that in the DR groups (Figure 8). These results showed that the application of melatonin greatly promoted the activities of antioxidant enzymes, and various enzymes played roles at different stages. Similarly, exogenous application of melatonin was shown to significantly increase the activity of the most important detoxifying enzymes in the leaves of drought-stressed apple [12], cucumber [14], and grape [15]. Furthermore, the total enzymatic activities of SOD and POD on 9 days correlated with the relevant gene transcript levels of *SOD [Cu-Zn]* and *POD12*, respectively, implying the conformity of the transcription and protein level (Figure 9). It may also be that the two genes mainly contributed to the synthesis of SOD and POD enzymes, separately. However, modulations in the total enzymatic activity of CAT did not correlate with the relevant transcript levels of two CAT isoenzyme genes (*CAT1, CAT6*), probably due to the organelles’ specificity or due to the involvement of other unknown genetic factors that may control the expression of these genes [37]. Hence, the results in the present study showed that melatonin can induce an increase in the antioxidant enzyme activities in kiwifruit leaves to remove excess ROS and protect against drought stress.

## 4. Materials and Methods

### 4.1. Plant Materials

The seeds of kiwifruit (*A. chinensis* var. deliciosa cv. Qinmei) were placed in a 4 °C refrigerator for 60 days in winter to break dormancy. After pregermination with poikilothermic treatment at 4 °C 10 h/ 25 °C 14 h for a week, the germinated seeds were cultivated in 25 °C for two weeks until they grew to two-ture-leaf stage. Then, the seedlings were transferred into plastic pots (diameter: 18 cm; height: 23 cm) filled with mixed soil consisting of peat substrate, pulverized coconut shell powder, and perlite (2:2:1), with three seedlings per pot. Then, all pots were moved to a greenhouse under natural light and temperature conditions at Sichuan Agricultural University, Chengdu, China (30°42′N, 103°51′E), following 2-day intervals with 1/2 Hoagland’s nutrient solution.

### 4.2. Experiment Design

Treatments began at 10-true-leaf stage, as follows: (1) Control (CK), plants were well-watered during the whole experimental time; (2) drought treatment (DR): seedlings were well-watered for 8 days, and subsequently irrigation was withheld for up to the next 9 days; and (3) melatonin and drought treatment (MTDR): Seedlings were pretreated with 50 mL 100 µM melatonin solution for 4 times in a two-day interval, then irrigation was withheld for up to next 9 days. Each treatment included 10 pots of seedlings and was repeated three times, and the day water withholding was begun was designed as 0 days. Their middle leaves (from five to eight per plant) were sampled after 0, 3, 6, and 9 days of drought treatment. A part of the collected samples were used for physiological indexes’ determination immediately, and others were immediately frozen in liquid nitrogen and stored at −80 °C for the following determination.

### 4.3. Measurement of Leaf Relative Water Content

The fresh weight (FW) was measured immediately after harvesting, and then saturated in water for 24 h. Thereafter, the saturated fresh weight (SW) was measured. The sample was dried in an oven at 105 °C for 15 min, and then was dried at 80 °C until the weight remained constant (DW). The relative leaf water content (WC) was calculated using the equation: WC = 100% × (FW − DW)/(SW − FW).

### 4.4. Determination of Hydrogen Peroxide (H_2_O_2_)

The H_2_O_2_ was extracted and colorimetrically measured as described by Zou (2000) [38]. In total, 0.3–0.5 g of leaves were ground as homogenate with 5 mL of cold acetone and centrifuged at 10,000× *g* for 10 min for 4 °C. Then, 1 mL of the extracted solution was mixed with 0.1 mL of 10% titanium chloride in HCl, 0.2 mL of strong aqua ammonia, and the mixture was then centrifuged. The precipitation was washed with acetone 3–5 times and 3 mL of 2 mol/L H_2_SO_4_ was added to dissolve the precipitation. The supernatant was measured at 415 nm by a spectrophotometer (Evolution 300, Thermo Fisher Scientific, Sunnyvale, CA, USA).

### 4.5. Assays for Lipid Peroxidation

Membrane damage was investigated by monitoring the MDA content, which was determined using the 2-thiobarbituric acid (TBA) test according to the method of Zou (2000) [38]. The homogenate was centrifuged at 16,000× *g* for 20 min at 4 °C. Two milliliters of 10% TCA containing 0.5% TBA was added to a 2-mL aliquot of the supernatant. The mixture was incubated in boiling water for 25 min and then cooled quickly in an ice bath, then centrifuged at 4000× *g* for 15 min. The content was measured at 532 nm and corrected for nonspecific absorbance at 600 nm by a spectrophotometer (Evolution 300, Thermo Fisher Scientific, Sunnyvale, CA, USA).

### 4.6. Estimation Metabolin Content and Enzyme Activity of the AsA-GSH Cycle

The contents of T-AsA, DHA, T-GSH, and GSSG were assayed following the method of our previous study [39]. The method for determination of T-AsA and AsA is based on the reduction of Fe^3+^ to Fe^2+^ by AsA in an acidic solution. The absorbance of the AsA and T-AsA reaction mixture was determined at 525 nm. The DHA content was calculated by deducting the reduced AsA content from the total ascorbate. For GSH and GSSH, the contents were determined at 412 nm. The GSH content was the difference between T-GSH and GSSG.

The activity of APX, DHAR, GR, and MDHAR was measured following Liang et al. [40]. In brief, 0.5 g of leaves were ground with 4% (*w*/*v*) PVPP, then homogenized with potassium phosphate buffer (pH 7.5) containing EDTA-Na_2_ and Triton X-100. The activity of APX was determined via monitoring of the absorbance decreases at 290 nm as reduced H_2_O_2_ was oxidized. MDHAR activity was assayed through monitoring of the absorbance decreases at 340 nm as NADH was oxidized. DHAR activity was determined through absorbance increases at 265 nm due to DHA formation. GR activity was assayed through absorbance decreases at 340 nm from NADPH oxidation.

### 4.7. Assays of Antioxidative Enzyme Activities

Frozen leaf sample (0.3 g) was ground with 25 mM HEPES buffer (pH 7.8) containing 0.2 mM EDTA, 2 mM AsA, and 2% PVP. The homogenates were centrifuged, and the resulting supernatants were used for the determination of enzymatic activity. Superoxide dismutase (SOD) activity was assayed by the nitroblue tetrazolium (NBT) method [41] based on photochemical reduction of nitro blue tetrazolium (NBT) as monitored at 560 nm. Activities of peroxidase (POD) and catalase (CAT) were assayed using the method of Scebba et al. [42]. POD was assayed at 470 nm by using hydrogen peroxide and guaiacol as the substrates. CAT activity was determined by monitoring the decrease in absorbance at 240 nm because of the decomposition of H_2_O_2_.

### 4.8. HPLC Analysis of Carotenoids

The carotenoids were extracted according to the method of Yan et al. [43]. Carotenoid components were measured using high-performance liquid chromatography (Agilent 1260, USA) equipped with a DAD detector. A C30 carotenoid column (250 × 4.6 mm, 5 µm) coupled to a C30 guard column (20 × 4.0 mm, 5 µm) (YMC Europe GMBH, Dinslaken, Germany) was used. The mobile phase was methanol:methyl tertiary butyl ether (7:3, *v*/*v*), flow rate was 0.5 × 10^−3^ L min^−1^, column temperature was 25 °C, and injection volume was 20 × 10^−6^ L. The carotenoid peaks were integrated at their individual maxima wavelength and their content were calculated using calibration curves of α-carotene, β-carotene, lutein, and zeaxanthin (Extrasynthese), respectively.

### 4.9. RNA Extraction and mRNA Sequencing

Total RNA was extracted from frozen flesh leaves using a modified CTAB method, and treated with RNase-free DNase I (Takara, Otsu, Shiga, Japan) to remove genomic DNA contamination. Sequencing libraries were generated from the three treatments (CK, DR, and MTDR) on 9 days with two biological replicates using an NEBNext^®^ Ultra™ RNA Library Prep Kit for Illumina^®^ (NEB, Ipswich, MA, USA) and sequenced on an IlluminaHiseq 2000 platform. HTSeq v0.6.1 was used to count the reads numbers mapped to each gene [44]. Then, FPKM (fragments per kilobase per million) of each gene was calculated based on the length of the gene and the reads count mapped to this gene. Differential expression analysis of two treatments was performed using the DESeq R package (1.18.0). The resulting *p*-values were adjusted using Benjamini and Hochberg’s approach for controlling the false discovery rate. Genes with an adjusted *p*-value < 0.05 found by DESeq were assigned as differentially expressed genes (DEGs) [43].

### 4.10. Validation by Quantitative Real-Time PCR (qRT-PCR)

The expression profiles of individual genes were assayed by RT-qPCR, performed on the CFX96 Real-Time System C1000 Thermal Cycler (Bio-RAD, Hercules, CA, USA) using a SYBR Premix Ex Taq kit (Takara, Otsu, Shiga, Japan) according to the manufacturer’s instructions, and analyzed using the 2^-ΔΔCT^ method. Relative gene expression was normalized by comparison with the expression of kiwifruit *actin* [45]. Details of the selected genes and the sequence of primers are listed in Table 3. The qRT-PCR experiments were repeated three times for three separate RNA extracts from three samples.

### 4.11. Statistical Analysis

Values are reported as means ± standard deviation (SD) from at least triplicate experiments. Data were analyzed using the software SPSS version 22.0 (IBM Corporation, Armonk, NY, USA). Differences between the means at the 5% level (*p* < 0.05) were considered statistically significant, which were established using one-way analysis of variance (ANOVA) followed by the least significant differences (LSD) test.

## Figures and Tables

**Table 1:** Summary of read numbers based on the RNA-Seq data.

Sample Name	Raw Reads	Clean Reads	Clean Bases (G)	Q20 (%)	GC Content (%)	Total Mapped (%)	Uniquely Mapped (%)
CK	47,300,498	45,707,847	6.86	97.15	45.90	68.56	66.16
DR	49,237,617	46,895,636	7.04	97.12	46.30	67.71	65.18
MTDR	54,138,373	51,348,163	7.71	97.26	45.86	68.33	65.74

**Table 2:** List of significant pathways identified by KEGG enrichments analysis of the annotated upregulated DEGs compared MTDR with DR.

KEGG ID	Pathway	Number of Transcripts	*p* Value
ath00480	Glutathione metabolism	24	0.0001
ath00053	Ascorbate and aldarate metabolism	12	0.0023
ath00906	Carotenoid metabolism	9	0.0057

**Table 3:** The sequences of primers in this study.

Gene Name	Forward Primer (5′→3′)	Reverse Primer (5′→3′)
*SOD*	AAAGGCGGGCTAGGGTTAGG	TGGAAGATCCGGGAGCGATA
*SOD[Cu-Zn]*	GCGGGTGACCTGGGAAACAT	AGGCTCTGCCGACGACTGAA
*POD12*	CTGCCCAGCACTAGACACAA	GTCCTGGTCGGACGTAAAAA
*POD42*	CCGAACGCGGTCCAGTATGT	TTGGTCCTCTTGTCGGTGGC
*CAT1*	ACCTGAGTGCCCTTTAAGCC	TTTGGGTATGAACGAGTTGG
*CAT6*	CTACATCCGCATCACCTTCG	TATCAGATTCGCTCCCGTCA
*APX4*	GCTGTCATGTCCGCATTCTT	GGTTTCTCGGCTTTGTTGGT
*APX6*	TCTTCACAGCTTTCGCATCT	AGCATTAGCACGGTATCCTT
*MDAR4*	GACTACCTGCCGTTCTTCTA	CTATCTCGCCTACACCATCT
*MDAR6*	CGCTGGAGAAAGCACAGAAA	GCGAGGGAAGGAGTAAACAA
*DHAR3*	AAAACATCTCCCTTACGACA	CTTTACCTTCTGGGCTTATT
*GR1*	CCTAATGAAGTCGAGGTGAC	GCCAGTTGCGATGAGTATGT
*AO*	CTCTACTCCAAGCCATTTCG	CTCCCTCTGACACTTACCG
*GME*	TTTGGCATTGAATGTAGG	AGGGTGTTGTCCGAGTTT
*VTC2*	CGCTACGATGTCACTGCT	CCTTGTCAACACGAAACTC
*MIOX4*	ATTTATGCGGAAGGCTGTG	GCCGAAGGTAGGGTGGTT
*MIOX5*	ATGATTGTATGGGTTGGA	GATGTCTGGGTTCTGCTC
*GSTL2*	GCCTGGTACAAGGAGAAA	CCAACAATAAGGGATGAAAT
*GSTU10*	TACAACCCAGTCCACAAA	CATTCCCTCTTCCACCAC
*GSTU18*	GTCCCTCCATCCTCCCTT	TCTCCTTGTGCCGTCCTA
*GSTU19*	TATGTTTGGGATGAGGGT	CTGGTAAGGGTCAGAGGG
*GSTU25*	GATACATAACGGAAAGCC	ACATAATCAGCCCAGAAC
*PSY*	TATAACGCTGCCTTGGCTCT	TTTCCGGCAAATATGTCCTC
*PDS*	CTTTGCATGCCAATAGCAGA	GTCGGACTTCACCACCAAGT
*LYC*	AGGAAGCAGCTGAAATCCAA	AGCCAGTCGCATCAAGAACT
*NPQ*	TTTTGCCTCTGAGCATTGTG	TCTACAAGGGGTGGTTCAGG
*CYP*	TAGCAAGCAACTCCGTGATG	ACTGCAGCTGATGTTTCGTG
*ZDS*	AGGAAGCAGCTGAAATCCAA	AGCCAGTCGCATCAAGAACT
*Actin*	TGCATGAGCGATCAAGTTTCAAG	TGTCCCATGTCTGGTTGATGACT

## Supplementary Materials

Supplementary materials can be found at https://www.mdpi.com/1422-0067/21/3/852/s1.
