# Supplementary material for: Melatonin Alleviates Drought Stress by a Non-Enzymatic and Enzymatic Antioxidative System in Kiwifruit Seedlings
Source: Int J Mol Sci. 2020 Jan 28;21(3):852. doi: 10.3390/ijms21030852 (PMC7036878; doi:10.3390/ijms21030852)
Supplement: Supplementary file 1 [file ijms-21-00852-s001.pdf]

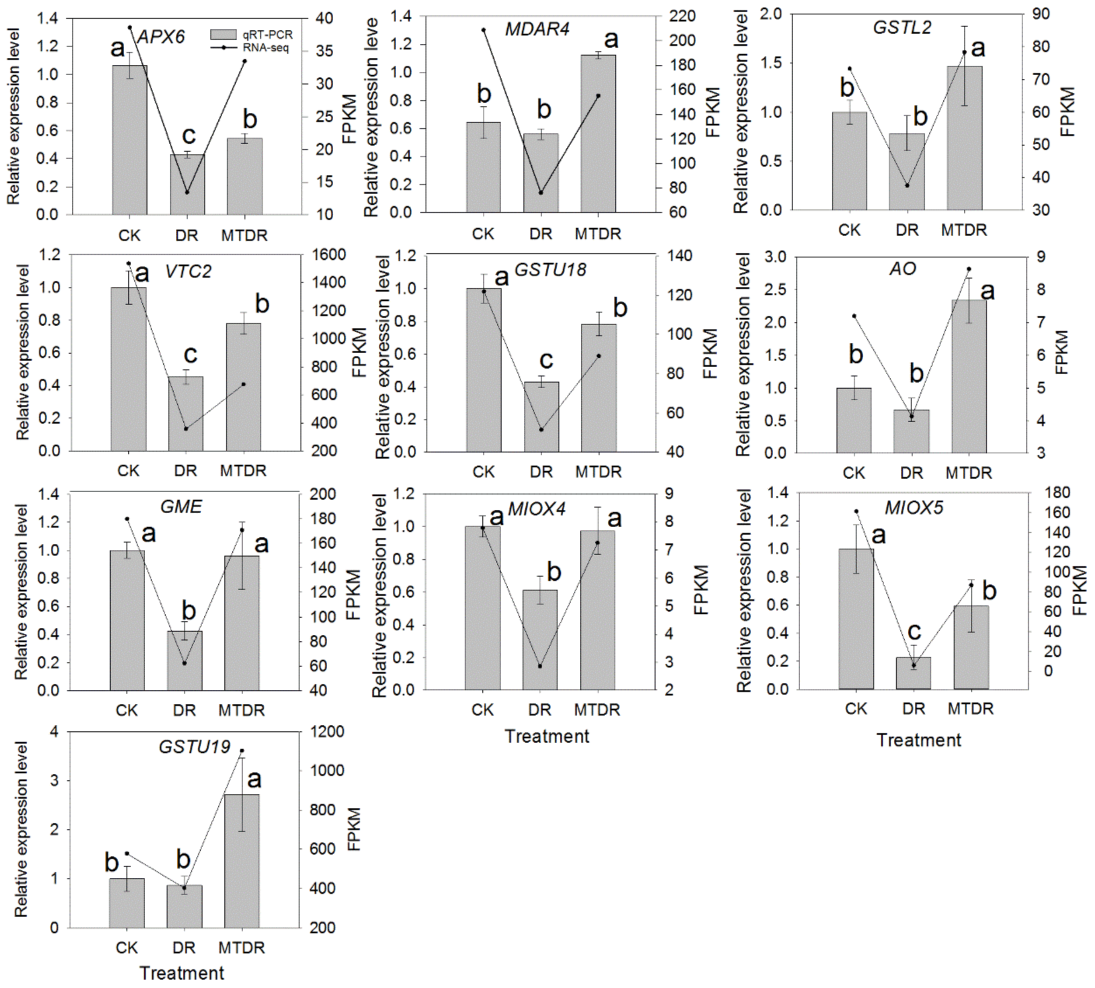

Supplemental material 1: Expression pattern of genes involved in AsA metabolism by RNA-Seq and qRT-PCR exposed to drought at 9d. Different treatments (x-axis) and gene expression (y-axis) by FPKM (right) and relative expression level (left). Gray columns in all plots indicate the relative expression level obtained by qRT-PCR; the black lines indicate the FPKM value obtained by RNA-seq. Bars represent the standard error (n=3). Different letters indicate significant differences according to LSD tests (P <0.05) for qRT-PCR.
